# A spatial covariance ^123^I-5IA-85380 SPECT study of α4β2 nicotinic receptors in Alzheimer's disease

**DOI:** 10.1016/j.neurobiolaging.2016.07.017

**Published:** 2016-11

**Authors:** Sean J. Colloby, Robert H. Field, David J. Wyper, John T. O'Brien, John-Paul Taylor

**Affiliations:** a Institute of Neuroscience, Newcastle University, Campus for Ageing and Vitality, Newcastle upon Tyne, UK; b Newcastle University Medical School, Newcastle University, Newcastle upon Tyne, UK; c SINAPSE, University of Glasgow, Institute of Neuroscience and Psychology, Glasgow, UK; d Department of Psychiatry, University of Cambridge, Cambridge, UK

**Keywords:** Alzheimer's disease, Cholinergic, Acetylcholine, Nicotinic, Spatial covariance, SPECT

## Abstract

Alzheimer's disease (AD) is characterized by widespread degeneration of cholinergic neurons, particularly in the basal forebrain. However, the pattern of these deficits and relationship with known brain networks is unknown. In this study, we sought to clarify this and used ^123^I-5-iodo-3-[2(S)-2-azetidinylmethoxy] pyridine (^123^5IA-85380) single photon emission computed tomography to investigate spatial covariance of α4β2 nicotinic acetylcholine receptors in AD and healthy controls. Thirteen AD and 16 controls underwent ^123^5IA-85380 and regional cerebral blood flow (^99m^Tc-exametazime) single photon emission computed tomography scanning. We applied voxel principal component (PC) analysis, generating series of principal component images representing common intercorrelated voxels across subjects. Linear regression generated specific α4β2 and regional cerebral blood flow covariance patterns that differentiated AD from controls. The α4β2 pattern showed relative decreased uptake in numerous brain regions implicating several networks including default mode, salience, and Papez hubs. Thus, as well as basal forebrain and brainstem cholinergic system dysfunction, cholinergic deficits mediated through nicotinic acetylcholine receptors could be evident within key networks in AD. These findings may be important for the pathophysiology of AD and its associated cognitive and behavioral phenotypes.

## 1 Introduction

One of the pathological characteristics of Alzheimer's disease (AD) is degeneration of cholinergic neurons of the basal forebrain (Davies and Maloney, 1976, Whitehouse et al., 1982) and acetylcholine is central to a myriad of brain functions including learning and memory (Hasselmo, 2006), attention (Himmelheber et al., 2000, Klinkenberg et al., 2011), and the sleep-wake cycle (Lee et al., 2005). As memory is a key cognitive domain that is impaired in AD, the cholinergic hypothesis was proposed as a key pathophysiological mechanism (Bartus et al., 1982). This remains at the heart of symptomatic management of AD, with widespread use of acetylcholinesterase inhibitors as principal first-line treatment, despite the variability in response and often limited benefits (Zemek et al., 2014). It is, therefore, important to continue to interrogate the role of cholinergic networks in AD in order to improve our understanding of these systems and to drive development of optimal therapeutic interventions in this condition.

Cholinergic neurotransmission in the brain is mediated by ionotropic nicotinic acetylcholine receptors (nAChRs) and metabotropic muscarinic receptors (mAChRs), both of which have been implicated in the cognitive deficits in AD (Petersen, 1977, Sarter and Paolone, 2011). The nAChR, which is the focus of this study, consists of 8α (α2–α7, α9, and α10) and 3β (β2–β4) subunits. These may assemble in different combinations to generate nAChR subtypes with varying electrophysiological properties and brain distribution (Albuquerque et al., 2009, Lindstrom et al., 1995), with the most abundant varieties in humans being α4β2 and α7.

There is now increasing evidence that decreased network connectivity is associated with aging and that this process is accelerated in AD, with specific systems such as the default mode network (DMN) particularly affected (Dennis and Thompson, 2014). Abnormalities of this and other distributed networks contribute to the AD symptomatology including memory changes (He et al., 2009, Sperling et al., 2010) and neuropsychiatric features (Rosenberg et al., 2015). However, what is less appreciated is the role the cholinergic system exerts on some of these key functional networks in AD. One way to examine network connectivity is by spatial covariance analysis. In AD, such procedures have previously been investigated with glucose metabolism positron emission tomography and perfusion single photon emission computed tomography (SPECT) imaging (Habeck et al., 2005, Johnson et al., 1998, Scarmeas et al., 2004). Indeed, we recently successfully applied the technique to ^123^I-iodo-quinuclidinyl-benzilate SPECT in AD, deriving an M1/M4 mAChR spatial covariance pattern (SCP) that characterized the receptor changes to cholinergic depletion (Colloby et al., 2015).

In the present study, we applied spatial covariance analysis to ^123^I-5IA-85380 SPECT scans, a ligand with high affinity for α4β2 nAChRs, in a sample of AD patients and healthy similar aged controls (O'Brien et al., 2007), to investigate disease-related α4β2 nAChR cholinergic networks. Since interpretation of ^123^I-5IA-85380 images in isolation is difficult due to uncertainty over the effects of cell loss, we also studied the corresponding perfusion resting-state networks.

## 2 Methods

### 2.1 Subjects

The sample consisted of 29 nonsmoking (>10 years) subjects (13 AD and 16 healthy elderly controls). Patients with AD were recruited from a community-dwelling population following referral to local old-age psychiatry services. Normal controls were recruited from friends and spouses of patients included in this and other research studies. All subjects underwent ^123^I-5IA-85380 and ^99m^Tc-exametazime SPECT scanning, with scans undertaken within 3 months of each other. The study was approved by the Newcastle, North Tyneside, and Northumberland local research ethics committee and the UK Department of Health's Administration of Radioactive Substances Advisory Committee (ARSAC). All participants and/or nearest relative (for patients who lacked capacity) gave informed written consent.

### 2.2 Assessments and diagnosis

Subjects underwent physical, neurological, and neuropsychiatric assessments, including mental state, history, physical examination, and for patients, blood screen with B12 and folate levels. The study battery administered included the Mini-Mental State Examination (MMSE) (Folstein et al., 1975), Neuropsychiatric Inventory (Cummings et al., 1994), Cambridge Cognitive Examination (CAMCOG) (Roth et al., 1986) with memory and executive function subscales (CAMCOG_memory_, CAMCOG_exec_).

Diagnosis was made by consensus between 2 experienced clinicians using the National Institute of Neurological and Communicative Disorders and Stroke/Alzheimer's Disease and Related Disorders Association (NINCDS/ADRDA) criteria for AD (McKhann et al., 1984). All AD subjects met criteria for probable AD. Controls had no signs or symptoms of cognitive disturbance and did not meet criteria for mild cognitive impairment, and all scored within the normal range of cognitive tests; ≥27 on MMSE, ≥90 on CAMCOG, and >2 standard deviations (SDs) above cutoff scores denoting cognitive impairment. Clinicopathological diagnosis was confirmed for 3 cases that subsequently died (1 control, 2 AD).

### 2.3 Radiochemistry

Radiosynthesis of ^123^I-5IA-85380 was produced form the corresponding stanyl precursor, 5-SnBu_3_-A85380, by electrophilic iododestanylation and performed according to details previously described (O'Brien et al., 2007).

### 2.4 Acquisition

Subjects were scanned with a triple-headed rotating gamma camera (Picker 3000XP), 2 hours post injection of 185 MBq of ^123^I-5IA-85380 using a previously reported imaging protocol (O'Brien et al., 2007). Within 3 months of ^123^I-5IA-85380 scanning, all subjects underwent ^99m^Tc-exametazime regional cerebral blood flow (rCBF) SPECT in accordance with methods described in an earlier study (Colloby et al., 2008).

### 2.5 Spatial preprocessing

All SPECT scans were spatially normalized to match, as appropriate, a ^123^I-5IA-85380 or ^99m^Tc-exametazime SPECT template in standard stereotactic space using linear image registration software (FLIRT: http://www.fmrib.ox.ac.uk/fsl/flirt/index.html). Generation of the template images has been described (Colloby et al., 2008, O'Brien et al., 2007). The spatially transformed images were then smoothed with a 10-mm full width at half maximum 3D Gaussian filter.

### 2.6 Spatial covariance analysis

Principal component (PC) analysis was applied on a voxel basis to all processed ^123^I-5IA-85380 SPECT images using covariance analysis software (http://www.nitrc.org/projects/gcva_pca/) (Habeck et al., 2005), producing a series of PC images. For each PC image, voxels had either positive or negative weights that represent the sign and strength of covariance between voxels. In this study, voxels with positive and negative weights were viewed as concurrently preserved/increased and decreased α4β2 binding, respectively. The extent to which an individual expressed the PC image was by way of a subject scaling factor (SSF) for that PC, calculated by superimposing the PC image onto an individual's processed ^123^I-5IA-85380 scan by computation of a “dot product,” which involves image multiplication on a voxel basis followed by summation of the products generating a score. Higher SSF scores for an individual for that PC image represents greater increased binding in voxels with positive weights and greater concurrent decreased binding in voxels with negative weights. To identify the ^123^I-5IA-85380 SCP that distinguished AD from controls, each individual SSF was entered into a linear regression model as explanatory variables with group as the dependent parameter. Akaike's information criteria (AICs) determined how many PCs should be included to reach optimal bias-variance trade-off (Burnham and Anderson, 2002). Seven PCs (1, 3, 4, 5, 6, 7, 10) yielded the lowest AIC value and were used to derive the SCP_5IA_. The degree to which each subject expressed the SCP_5IA_ was by way of the SSF_5IA_.

The same approach was applied to the ^99m^Tc-exametazime SPECT scans. Therefore, positive and negative weights were interpreted as concurrent increased and decreased rCBF, respectively. Five PCs (1, 2, 3, 4, 12) yielded the lowest AIC value and were used to generate the SCP_rCBF_ that best separated AD from controls, while each subject expressed the SCP_rCBF_ by their SSF_rCBF_.

Stability and reliability of the SCPs were assessed by bootstrap resampling (1000 iterations), to identify areas that contributed to the patterns with high confidence. This transforms the voxel weights of each SCP into Z maps, computed as the ratio of voxel weight and bootstrap standard deviation. The Z-statistic follows roughly a standard normal distribution where a one-tailed *p* ≤ 0.05 infers a threshold of |Z| ≥ 1.64 (Habeck et al., 2010). Anatomical labeling of the Z maps was performed using the image visualization software “FSLView” (http://fsl.fmrib.ox.ac.uk/fsl/fslview/), which contains various anatomical brain atlases from which the labels were reported from.

### 2.7 Statistical analyses

Continuous variables were tested for normality using visual inspection of histograms and Shapiro-Wilk test. Demographic, clinical, and imaging measures were assessed, where applicable, using parametric (analysis of variance) and nonparametric χ^2^ tests. Correlations were performed using Pearson's r coefficients. Statistical tests were interpreted as significant if *p* ≤ 0.05. Data analysis used the Statistical Package for Social Sciences software (SPSS version 22.0, http://www-01.ibm.com/software/analytics/spss/products/statistics/).

## 3 Results

### 3.1 Subject demographics and clinical characteristics

Patient demographic and clinical characteristics are reported in Table 1. AD subjects and controls were similar with respect to gender, although there was a small difference in age with AD patients being slightly older than controls. As expected, AD subjects were impaired on all cognitive measures compared to controls (*p* < 0.001). Two patients were receiving cholinesterase treatment (donepezil, standard daily clinical dose 10 mg) (for >3 months) at the time of the study.

### 3.2 Spatial covariance

**Fig. 1:**
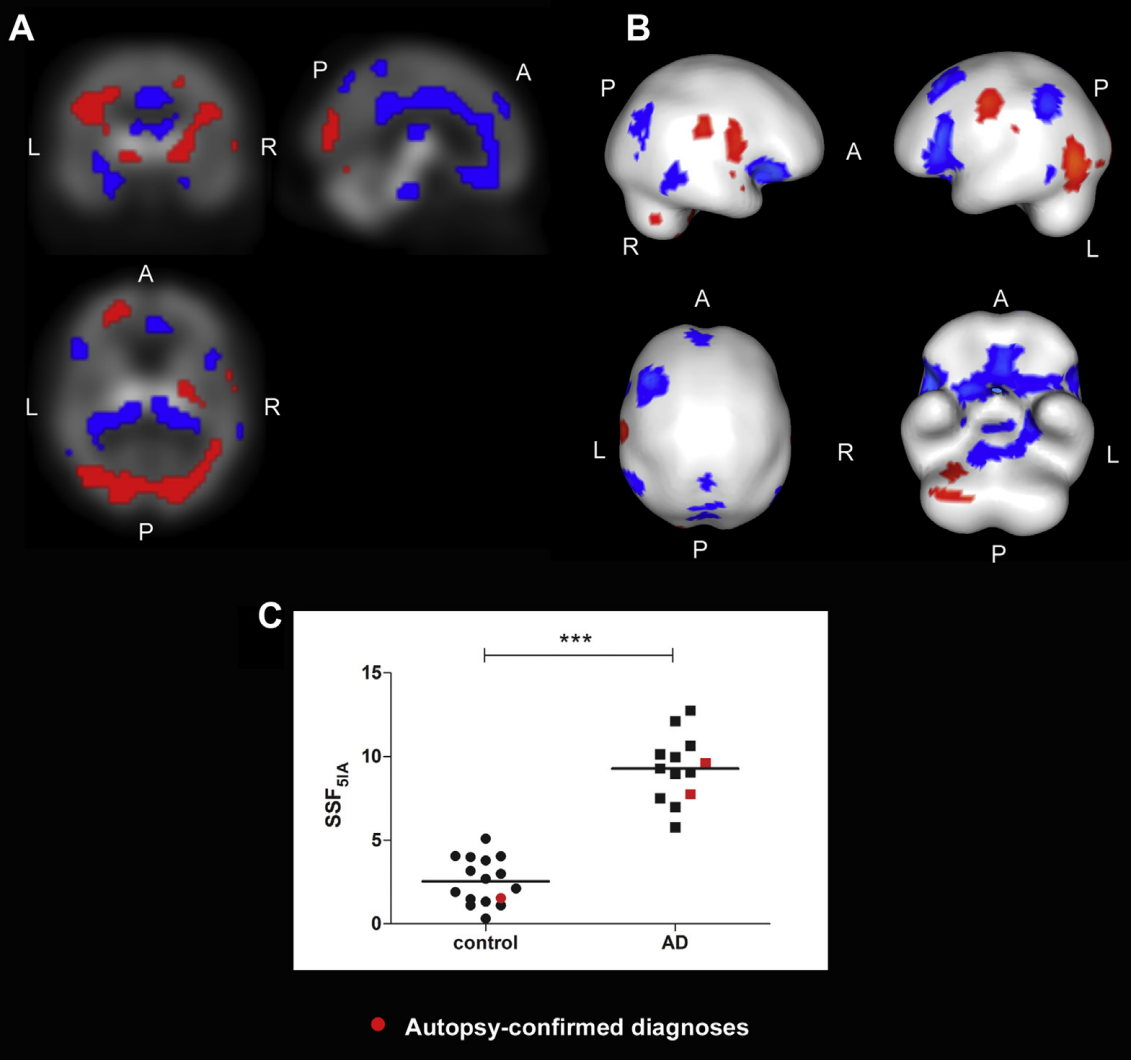
α4β2 nicotinic acetylcholine receptor spatial covariance pattern in AD (|Z| ≥ 1.64, *p* ≤ 0.05). Orthogonal (A) and rendered (B) views of the α4β2 nAChR spatial covariance pattern superimposed upon the ^12^^3^I-5IA-85380 SPECT template distinguishing AD from controls. Red and blue regions depict relative increased and/or preserved and decreased activity, respectively. (C) Distribution of SSF_5IA_ scores in controls (n = 16) and AD (n = 13) (***p < 0.001). Abbreviations: A, anterior; AD, Alzheimer's disease; L, left; nAChR, nicotinic acetylcholine receptor; P, posterior; R, right; SSF, subject scaling factor. (For interpretation of the references to color in this figure legend, the reader is referred to the Web version of this article.)

The α4β2 nAChR voxel SCP_5IA_ that distinguished AD from controls is shown in Fig. 1A and B. SSF_5IA_ scores, representing the extent to which subjects expressed the topography, were higher in AD than controls (mean ± SD; AD = 9.3 ± 2.0, controls = 2.6 ± 1.4, t_27_ = 10.8, *p* < 0.001, Fig. 1C). The pattern was mainly characterized by relative decreases in α4β2 binding (blue) in basal forebrain, pedunculopontine, thalamus, limbic, parietal, and frontal regions together with relative preserved or increased binding (red) in midbrain, pallidum, cerebellum, occipital, and pre/post central gyri. Table 2 depicts details of specific regions contributing to the α4β2 disease-related pattern with high confidence (|Z| ≥ 1.64, *p* ≤ 0.05).

**Fig. 2:**
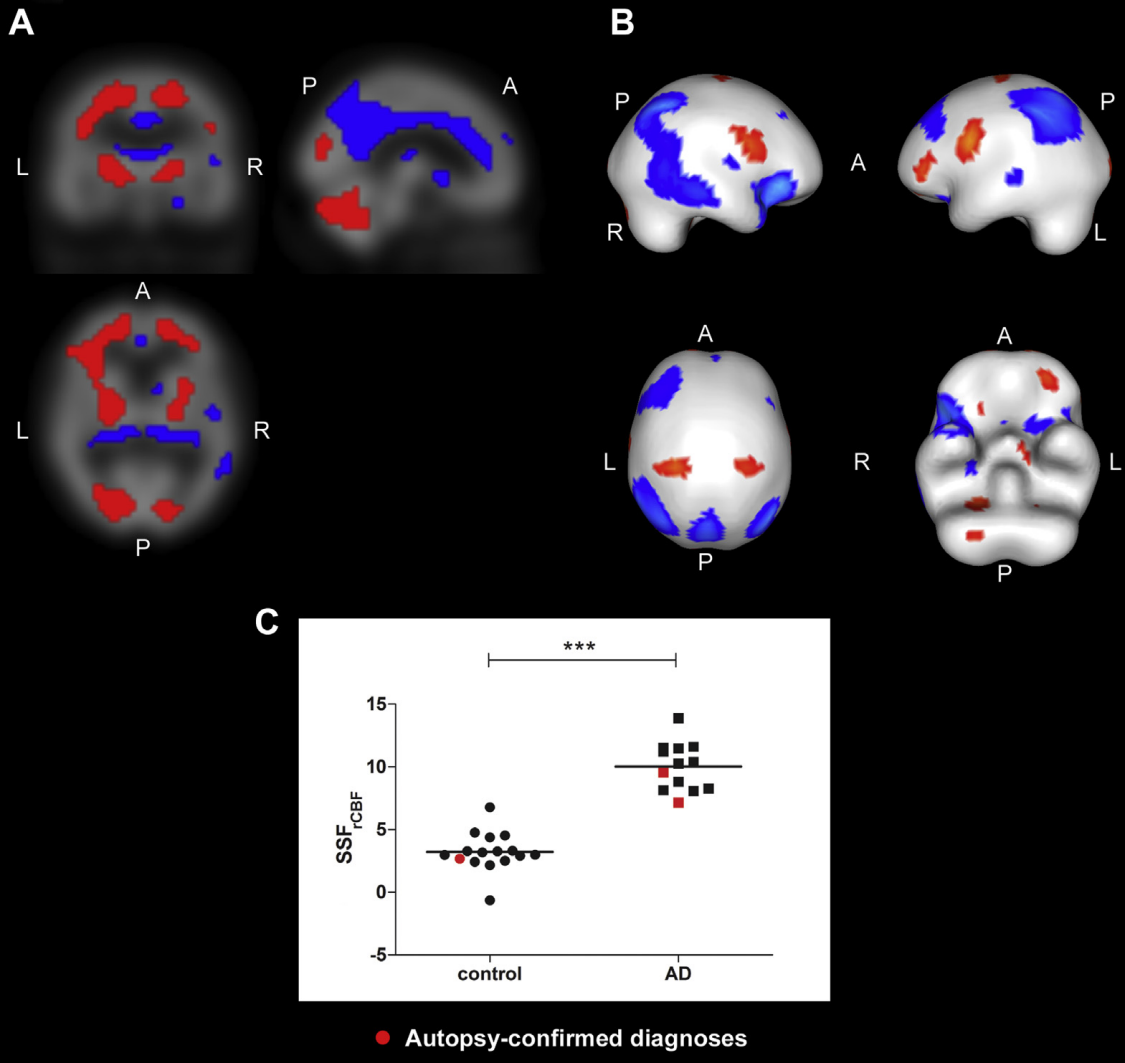
Regional cerebral blood flow spatial covariance pattern in AD (|Z| ≥ 1.64, *p* ≤ 0.05). Orthogonal (A) and rendered (B) views of the rCBF spatial covariance pattern superimposed upon the ^99m^Tc-exametazime SPECT template distinguishing AD from controls. Red and blue regions depict relative increased and decreased activity respectively. (C) Distribution of SSF_rCBF_ scores in controls (n = 16) and AD (n = 13) (***p < 0.001). Abbreviations: A, anterior; AD, Alzheimer's disease; L, left; P, posterior; R, right; rCBF, regional cerebral blood flow; SSF, subject scaling factor. (For interpretation of the references to color in this figure legend, the reader is referred to the Web version of this article.)

The associated rCBF SCP_rCBF_ that differentiated AD from controls is depicted in Fig. 2A and B, where SSF_rCBF_ scores differed between groups (mean ± SD; AD = 10.0 ± 1.9, controls = 3.2 ± 1.5, t_27_ = 10.6, *p* < 0.001, Fig. 2C). The pattern mainly comprised of relative decreased rCBF (blue) in thalamus, cingulate, parietal, and prefrontal areas with relative increases (red) in cerebellum, lentiform nucleus, lingual gyrus, and precentral regions. Table 3 depicts details of specific regions significantly contributing to the rCBF disease–related pattern (|Z| ≥ 1.64, *p* ≤ 0.05).

Relationship between SCP expressions and age, MMSE, CAMCOG, CAMCOG_memory_, and CAMCOG_exec_ were investigated in AD. Trends were observed in CAMCOG (r = −0.52, *p* = 0.03), CAMCOG_memory_ (r = −0.51, *p* = 0.04), and MMSE (r = −0.54, *p* = 0.03) with SSF_5IA_ but not age and CAMCOG_exec_ (|r| ≤ 0.37, *p* ≥ 0.11). For SSF_rCBF_, CAMCOG (r = −0.47, *p* = 0.05) and MMSE (r = −0.52, *p* = 0.04) correlated, while for all other measures (|r| ≤ 0.42, *p* ≥ 0.08).

**Fig. 3:**
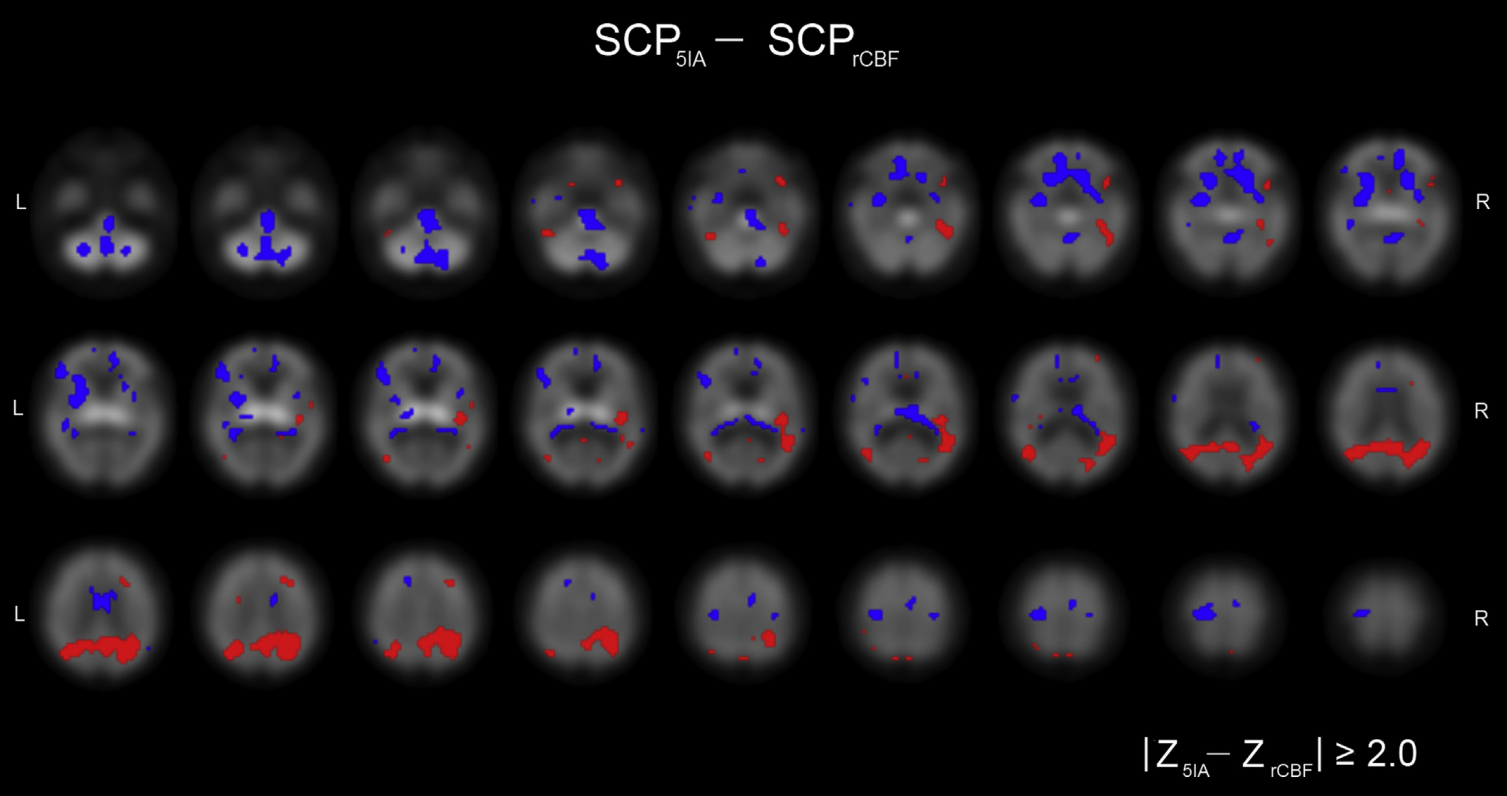
Difference image between the nicotinic and regional cerebral blood flow spatial covariance patterns displayed axially and superimposed upon the ^123^I-5IA-85380 SPECT template. Red and blue regions represent (Z_5IA_ − Z_rCBF_) ≥ 2.0 and (Z_5IA_ − Z_rCBF_) ≤ −2.0, respectively. Abbreviations: L, left; R, right; rCBF, regional cerebral blood flow; SCP, spatial covariance pattern. (For interpretation of the references to color in this figure legend, the reader is referred to the Web version of this article.)

It was evident that there was some degree of similarity between the nicotinic and rCBF patterns, supported by the significant correlations that were observed between the SSF_5IA_ and SSF_rCBF_ scores in controls (r = 0.61, *p* = 0.006) and AD (r = 0.74, *p* = 0.002). We, therefore, sought to explore in a descriptive sense, regions that differed between the 2 patterns by subtraction of their respective bootstrapped spatial covariance Z maps. Fig. 3 shows the difference in image (SCP_5IA_ − SCP_rCBF_), where the blue and red regions represent (Z_5IA_ − Z_rCBF_) ≤ −2.0 and (Z_5IA_ − Z_rCBF_) ≥ 2.0, respectively. The difference image showed deviations in pedunculopontine nucleus, insula, anterior cingulate, putamen, thalamus, and frontal regions (blue) along with precuneus, lateral occipital, temporal, and parietal areas (red). Blue regions characterize greater negative weights in the nicotinic pattern relative to rCBF or where voxel weightings are of opposite sign (nicotinic −ve, rCBF +ve), whereas red regions describe greater positive weights in the nicotinic pattern relative to rCBF or where voxel weightings are of opposite sign (nicotinic +ve, rCBF −ve). Table 4 presents details of specific regions contributing to the difference pattern.

## 4 Discussion

We undertook a multivariate network perspective of ^123^I-5IA-85380 SPECT, a α4β2 nAChR ligand in AD. We derived disease-related α4β2 nAChR and rCBF patterns of spatial covariance, which implies the presence of several dysfunctional cholinergic and perfusion networks in AD. These findings represent the first attempt to differentiate AD from controls through spatial covariance analysis of cholinergic nicotinic receptor activity and/or availability, and follow our multivariate assessment of M1/M4 mAChR binding in AD (Colloby et al., 2015).

The nAChR covariance pattern comprised of decreased and preserved and/or increased activity in a number of concomitant brain areas. The covariant negative-weighted pattern converged on various subcortical and neocortical regions, implicating a number of cholinergic networks. In particular, the basal forebrain–neocortex and hippocampus system appeared to be affected, and this network is widely recognized as a significant contributor to memory and learning processes. The brainstem system which may mediate the sleep-wake cycle also appears to be involved with relative reduced uptake in pedunculopontine nucleus and thalamus. This observation is in keeping with clinical observations that sleep-wake disturbances are highly prevalent and often a disabling feature in AD (Lim et al., 2014), although in the present study we did not have data on the nature or severity of any sleep disturbances in our patient group to clarify any specific clinical relationships. We also observed a relative decreased binding which mapped onto DMN hubs, namely, medial prefrontal, posterior cingulate, precuneus, and inferior parietal. This network is active during rest and deactivates during goal-directed behaviors (Raichle et al., 2001), where evidence has shown reduced DMN activity in AD (Greicius et al., 2004, Simic et al., 2014) and its contribution to cognitive decline (Seeley et al., 2009). One study reported convergences of amyloid deposition, metabolic disruption, and atrophy of the DMN in AD (Buckner et al., 2005), suggesting that the relative decreased pattern within this network could be in fact characterizing these pathological and/or functional deficits, although an element of concomitant DMN cholinergic dysfunction cannot be excluded. Reduced DMN activity of nAChRs was consistent with our previous findings of reduced M1/M4 mAChR expressions within similar regions (Colloby et al., 2015), highlighting the potential role of both types of receptors in AD and that the cholinergic system may have a more fundamental role in the normal functioning of the DMN. Other mappings onto established resting-state networks, included the anterior insula and anterior cingulate, which are key nodes of the “salience network,” for initiation of cognitive control and switching networks to aid access to working memory and attention resources (Menon and Uddin, 2010, Seeley et al., 2009). Networks involving the insula have also been shown to play a role in episodic memory (Xie et al., 2012). Furthermore, regions also mapped onto the Papez circuit, a limbic network incorporating the thalamus, hippocampus, and cingulate cortex (Papez, 1937). Its function lies in emotional processing and memory, where numerous studies have demonstrated its dysfunction and atrophy in AD (Allen et al., 2007, Jones et al., 2006, Smith, 2002).

The associated rCBF pattern largely comprised of relative decreases in thalamus, cingulate, parietal, and prefrontal areas with relative increases in cerebellum, lentiform nucleus, lingual gyrus, and precentral regions, indicating impairment in a number of functional networks. Regions that were concomitantly reduced appear to involve hubs of the DMN and frontoparietal attention (inferior parietal, dorsolateral prefrontal cortex) networks (Markett et al., 2014); the latter finding chimes with a recent study depicting dorsal and ventral attention systems dysfunction in AD and amnestic mild cognitive impairment (Zhang et al., 2015). The covariant pattern was also broadly consistent to previous spatial covariance studies in AD using H_2_^15^O positron emission tomography (Scarmeas et al., 2004) and arterial spin labeling perfusion magnetic resonance imaging (Asllani et al., 2008). Differences in results between these studies were likely attributed to variations in image modality and/or in AD populations.

As an exploratory analysis, we examined correlations between the cholinergic and perfusion covariance pattern expressions and measures of cognition in AD. Although results were not corrected for multiple tests, negative trends were observed with CAMCOG and MMSE scores, suggesting the cholinergic and/or perfusion patterns and the various networks they represented were similarly related to global cognition and/or dementia severity. Memory scores (CAMCOG_memory_) only appeared to be associated with the cholinergic pattern which is not unexpected given the observed deficits to spatial networks involving the medial temporal lobe structures, and the putative role of acetylcholine on learning and memory (Hasselmo, 2006, McGaughy et al., 2000), as well as the established therapeutic efficacy of cholinesterase inhibitors in ameliorating cognitive symptoms in AD (Zemek et al., 2014). Although our AD group was relatively small, these results highlight that a larger-scale covariance study may be useful to investigate specific correlates between the clinical phenotype of AD and cholinergic network dysfunction.

Although we used stratified bootstrap resampling to verify the stability and reliability of the SCPs, the results were nonetheless obtained from small samples and as a consequence may not be generalizable and as such were a major drawback of this study. Another study limitation was a minority of autopsy-confirmed diagnoses and a relatively moderate demented patient group. It was also difficult to establish whether the observed cholinergic networks were attributed exclusively to changes in nicotinic receptor expression or to some extent by the functional rCBF changes. Although descriptive and not rigorously inferential, we investigated differences between the nicotinic and rCBF patterns in order to identify areas of relative variation. The deviation map suggests that brainstem system and salience networks were perhaps more associated with nicotinic receptor expression, whereas for DMN and Papez circuits suggest that this was less clear. Receptor availability and rCBF were also influenced by atrophy and partial volume effects, so both types of scans were likely to be equally affected by neural degeneration. Thus, common regions of relative decreased uptake that are susceptible to AD pathology may indicate atrophic effects rather than receptor or functional changes. In addition, although there was a small age difference between the groups, there were no significant correlations between age and the nicotinic and rCBF subject scores in controls or AD and therefore, unlikely to affect the discriminant patterns. Strengths of this study were in examining AD patients that were mainly free from any cholinergic medications as well as having both nicotinic and perfusion SPECT scans available for all subjects.

## 5 Conclusions

The multivariate perspective provides further insights into the pathophysiological changes in AD. The SCP not only suggests basal and brainstem cholinergic system deficits, but DMN, salience and Papez cholinergic circuits may also be vulnerable, and that the cholinergic system might have a more fundamental role in the normal functioning of these networks in AD. Future studies could examine the cholinergic network profiles that are associated with positive treatment outcomes from therapies aimed at improving cholinergic neurotransmission.

## Disclosure statement

Dr Colloby, Dr Field, and Professor Wyper report no disclosures. Professor O'Brien has been a consultant for GE Healthcare, Lilly, Bayer Healthcare, TauRx, and Nutricia and has received honoraria for talks from GE Healthcare, Lilly, and Novartis. Dr Taylor has been a consultant of Lundbeck and received honoraria for talks from GE Healthcare and Flynn pharmaceuticals.

## Figures and Tables

**Table 1:** Demographic, clinical, and neuropsychological information for individuals studied with^123^I-5IA-85380 SPECT

	Control	AD	Statistic, *p* value
N	16	13	
Gender (male:female)	10:6	6:7	χ^2^_(1)_ = 0.8, *p* = 0.4
Age	75.4 ± 4.5	79.7 ± 6.6	**F**_**1,27**_**= 4.5, *p* = 0.04**
MMSE	28.8 ± 1.0	17.7 ± 5.2	**F**_**1,27**_**= 69.8, *p* < 0.001**
CAMCOG	97.8 ± 3.8	59.8 ± 19.4	**F**_**1,27**_**= 59.0, *p* < 0.001**
CAMCOG_memory_	24.0 ± 1.8	10.2 ± 5.9	**F**_**1,27**_**= 79.0, *p* < 0.001**
CAMCOG_exec_	23.3 ± 3.1	10.8 ± 4.0	**F**_**1,27**_**= 90.5, *p* < 0.001**
NPI	n/a	12.2 ± 12.3	
Medications			
ChEIs	0	2	

Data are expressed as mean ± 1 SD.

Bold text denotes significant differences.

Key: AD, Alzheimer's disease; CAMCOG, Cambridge Cognitive Examination; CAMCOG_exec_, executive function component of CAMCOG; CAMCOG_memory_, memory component of CAMCOG; ChEIs, cholinesterase inhibitors; MMSE, Mini-Mental State Examination; n/a, not applicable; NPI, Neuropsychiatric Inventory; SD, standard deviation.

**Table 2:** Regions contributing to the α4β2 disease–related pattern with high confidence (|Z| ≥ 1.64, *p* ≤ 0.05) in AD

Hemisphere	MNI coordinates	Region	Z score
	4, −29, −28	Pedunculopontine nucleus	−2.2
L	−8, 8, −12	Nucleus accumbens	−1.9
R	12, 8, −12	Nucleus accumbens	−2.0
L	−24, −12, −24	Hippocampus	−2.3
R	28, −12, −20	Hippocampus	−1.7
L	−20, −4, −20	Amygdala	−1.9
R	28, 0, −20	Amygdala	−1.8
L	−16, 16, −20	Orbitofrontal cortex	−1.9
R	24, 12, −20	Orbitofrontal cortex	−2.3
L	0, 36, −16	Medial frontal cortex	−2.1
R	4, 36,−16	Medial frontal cortex	−2.0
L	−28, 20, −8	Insula	−1.7
R	44, 12, −4	Insula	−2.5
L	−52, 24, 4	Inferior frontal gyrus	−2.0
R	56, 16, 4	Inferior frontal gyrus	−2.2
L	−8, −20, 16	Thalamus	−2.5
R	12, −20, 16	Thalamus	−2.9
L	−4, 8, 36	Anterior cingulate	−3.3
R	4, 32, 16	Anterior cingulate	−2.3
L	0, −44, 32	Posterior cingulate	−2.0
R	4, −32, 32	Posterior cingulate	−1.8
L	−48, −48, 44	Inferior parietal	−2.2
R	52, −60, 48	Inferior parietal	−1.7
L	−4, −72, 52	Precuneus	−1.9
R	4, −48, 64	Precuneus	−1.9
R	16, 8, −8	Putamen	−1.9
L	−12, −60, −36	Posterior cerebellum	2.4
R	20, −56, −36	Posterior cerebellum	2.8
L	−12, −60, −24	Anterior cerebellum	2.3
R	20, −56, −24	Anterior cerebellum	2.6
L	−12, −16, −10	Midbrain	2.0
R	18, −18, −10	Midbrain	2.0
L	−20, −12, 0	Pallidum	2.1
R	24, −12, 0	Pallidum	2.0
L	−40, −72, 12	Middle occipital gyrus	2.5
R	36, −84, 16	Middle occipital gyrus	2.9
L	−12, −88, 16	Cuneus	2.8
R	20, −84, 16	Cuneus	2.7
L	−48, −16, 32	Postcentral gyrus	2.2
R	52, −12, 32	Postcentral gyrus	2.5
L	−44, −4, 36	Precentral gyrus	2.0
R	52, −4, 36	Precentral gyrus	1.8

Key: AD, Alzheimer's disease; L, left; MNI, Montreal Neurological Institute; R, right.

**Table 3:** Regions contributing to the corresponding rCBF pattern with high confidence (|Z| ≥ 1.64, *p* ≤ 0.05) in AD

Hemisphere	MNI coordinates	Region	Z score
L	−60, −40, 0	Middle temporal gyrus	−1.9
R	68, −32, −16	Middle temporal gyrus	−1.9
L	−12, −28, 12	Thalamus	−2.4
R	16, −28, 12	Thalamus	−2.3
L	−4, −48, 20	Posterior cingulate	−2.2
R	4, −48, 28	Posterior cingulate	−2.6
L	0, 20, 28	Anterior cingulate	−2.2
R	4, 20, 28	Anterior cingulate	−2.8
L	0, −56, 36	Precuneus	−4.0
R	4, −60, 32	Precuneus	−3.9
L	−40, −64, 44	Inferior parietal	−2.4
R	48, −64, 44	Inferior parietal	−2.9
L	−36, 20, 52	Middle frontal gyrus	−2.1
R	32, 32, 44	Middle frontal gyrus	−1.7
L	−24, 28, 52	Superior frontal gyrus	−2.2
L	−16, 16, −24	Inferior frontal gyrus	−1.9
L	−16, −64, −36	Posterior cerebellum	4.2
R	24, −64, −36	Posterior cerebellum	3.8
L	−16 −64, −28	Anterior cerebellum	3.2
R	24, −64, −28	Anterior cerebellum	3.8
L	−20, 16, −8	Putamen	2.2
R	24, 8, −4	Putamen	1.7
L	−20, −4, −4	Pallidum	2.1
R	24, −12, 0	Pallidum	1.8
L	−56, 0, 24	Precentral gyrus	2.2
R	60, 4, 24	Precentral gyrus	1.8
L	−16, 48, 4	Medial frontal gyrus	2.5
R	20, 44, 4	Medial frontal gyrus	2.3
L	−20, −56, −12	Lingual gyrus	2.0
R	20, −56, −12	Lingual gyrus	2.0

Key: AD, Alzheimer's disease; L, left; MNI, Montreal Neurological Institute; R, right; rCBF, regional cerebral blood flow.

**Table 4:** Regions (|Z_5IA_ − Z_rCBF_| ≥ 2.0) contributing to the difference in image (SCP_5IA_ − SCP_rCBF_)

Hemisphere	MNI coordinates	Region	Z_5IA_ − Z_rCBF_
	8, −28, −28	Pedunculopontine nucleus	−3.2
R	4, −60, −36	Vermis cerebellum	−3.1
R	28, −68, −36	Posterior cerebellum	−2.4
L	−24, −64, 36	Posterior cerebellum	−3.3
R	40, 0, −4	Insula	−2.5
L	−32, 24, 0	Insula	−2.1
R	24, 16, −8	Putamen	−3.7
L	−20, 16, −8	Putamen	−3.6
R	4, 8, 32	Anterior cingulate	−2.8
L	0, −4, 36	Anterior cingulate	−2.9
R	28, 20, −12	Orbitofrontal cortex	−3.2
L	−28, −8, −16	Amygdala	−2.8
L	0, 24, −12	Subcallosal cortex	−2.6
L	0, 44, −12	Medial frontal cortex	−2.3
L	−16, 4, 0	Pallidum	−2.1
L	−16, −24, 4	Thalamus	−2.4
L	−52, 32, 8	Inferior frontal gyrus	−3.1
L	−8, 36, 28	Paracingulate gyrus	−2.5
L	−20, −20, 64	Precentral gyrus	−2.7
L	0, −60, 32	Precuneus	2.7
R	8, −56, 32	Precuneus	3.1
L	−44 −68, 16	Lateral occipital cortex	2.4
R	48, −64, 20	Lateral occipital cortex	2.2
R	56, −44, −16	Inferior temporal gyrus	2.2
R	52, 16, −12	Temporal pole	2.6
R	60, −52, 16	Angular gyrus	2.7
R	36, −60, 48	Superior parietal lobe	3.5

Key: L, left; MNI, Montreal Neurological Institute; R, right; rCBF, regional cerebral blood flow; SCP, spatial covariance pattern.
